# Effects of a wearable-based intervention in overweight and obese adolescents: A randomized controlled trial considering gender, baseline activity, and intervention exposure

**DOI:** 10.1017/wtc.2026.10039

**Published:** 2026-03-30

**Authors:** Adrián Mateo-Orcajada, Raquel Vaquero-Cristóbal, Lucía Abenza-Cano

**Affiliations:** 1 Research Group Movement Sciences and Sport (MS&SPORT), Department of Physical Activity and Sport, Faculty of Sport Sciences, University of Murcia, Murcia, Spain; 2 Facultad de Deporte, UCAM, Universidad Católica San Antonio de Murcia, Murcia, Spain

**Keywords:** adolescents, body composition, physical fitness, randomized controlled trial, wearables

## Abstract

The objectives of the present study were (a) to determine the effects of a 12-week intervention using wearables promoted through physical education classes on physical activity, body composition, physical fitness and psychological well-being of overweight or obese adolescents; and (b) to analyze the differences in outcomes based on gender and baseline physical activity. Seventy-three overweight and obese adolescents (mean age: 13.44 ± 1.12 years) were randomly assigned to an experimental group (EG) or control group (CG). The EG used a physical activity wearable for 12 weeks. Both groups were assessed before and after the intervention. Regarding primary outcomes, the EG showed an increase in physical activity (*p* = 0.048) and reductions in body mass index (*p* = 0.007), fat mass (*p* < 0.001), and sum of 3 skinfolds (*p* = 0.002), with moderate-to-large effect sizes (*η*^2^ > 0.09). According to the secondary outcomes, improvements in physical fitness were limited, with increases observed only in abdominal muscular endurance, and these changes were also present in the CG. Subgroup analyses showed that females and adolescents with low baseline physical activity experienced greater benefits, particularly in fat-related variables (*p* < 0.001–0.037), with large effect sizes (*η*^2^ > 0.14). Additionally, adolescents with greater exposure to the wearable-based intervention showed more consistent improvements in fat-related outcomes (*p* < 0.001–0.032), with large effect sizes (*η*^2^ > 0.25). In conclusion, a wearable-based intervention promoted through physical education classes may contribute to meaningful improvements in body composition, particularly among females and previously inactive adolescents who are overweight or obese. However, effects on physical fitness and psychological well-being were limited, highlighting the importance of intervention design, adherence, and complementary motivational strategies.

## 1. Introduction

Engaging in physical activity during adolescence is essential for the prevention and treatment of chronic conditions such as being overweight and obesity (Wellman et al., 2020). In this context, meeting the World Health Organization recommendations of at least 60 minutes of daily moderate-to-vigorous physical activity is crucial (O’Donovan et al., 2010). However, approximately 80% of adolescents discontinue regular physical activity during early adolescence (Pechtl et al., 2022). In addition, sedentary behaviors have increased in recent years by 3.4% in males and 5.3% in females (Kong et al., 2021), leading to lifestyle changes that negatively impact adolescents’ physical, social, and mental health (Quazi and Tankha, 2023).

Previous research has shown that increasing daily step counts can improve the body composition, physical fitness, and psychological well-being of adolescents (Stojanović et al., 2024). These benefits appear to be particularly relevant for adolescents who are overweight or obese, as walking enhances physiological activation and energy expenditure (Murtagh et al., 2014). Nevertheless, this population is difficult to reach through interventions due to very low adherence rates, resulting in few participants completing the programs (Peral-Suárez et al., 2020).

Traditional physical activity interventions, generally based on structured exercise programs or organized sports, are not attractive or accessible to all adolescents, particularly those who are overweight or obese, which reduces their adherence to these types of programs (Stankov et al., 2012; Chen et al., 2024). Barriers such as perceived low competence, previous negative experiences with physical activity, or limited autonomy can reduce motivation and long-term commitment in this population (Chen et al., 2024; Stankov et al., 2012). Moreover, school-based physical activity opportunities are limited, as most curricula include only two physical education sessions per week, which is insufficient to promote long-term healthy habits (Hills et al., 2015).

As a result, there is a growing need for approaches that promote physical activity in a more flexible way, allowing adolescents to progress at their own pace in order to be as inclusive as possible, integrating movement into adolescents’ daily routines rather than relying exclusively on formal exercise settings (Corder et al., 2015; Ulset et al., 2025). Among these, technology-based approaches, particularly mobile applications, have gained increasing attention as tools to promote physical activity engagement (Bosworth et al., 2023). These technologies are appealing because they can be used both outside school hours (Mateo-Orcajada et al., 2023) and during physical education classes.

Previous studies have shown that their use in school settings can increase daily step counts and overall physical activity levels (Zhu and Dragon, 2016; Schoeppe et al., 2017). However, the integration of mobile technologies into physical education classes has also been criticized in school contexts (Thorburn and Stolz, 2017). As a result, several studies have focused on evaluating their effectiveness when implemented outside school hours, particularly among adolescents who are overweight or obese (Cummings et al., 2022; Mateo-Orcajada et al., 2024).

Despite observed improvements in physical fitness and body composition, a decline in adherence to interventions using these devices is commonly reported after the initial weeks of use (He et al. 2021). This decrease is attributed to the complexity of mobile application functionalities, which can affect adolescents’ perceived usability (Bhandari et al., 2017; Bardus et al., 2019). In addition, limited compatibility between mobile applications and different devices may further reduce the effectiveness of these interventions (Alonso-Fernández et al., 2022).

To address these limitations, wearable devices such as smartwatches and activity trackers have been increasingly promoted. These devices allow for the objective monitoring of daily step counts and have shown potential for increasing physical activity and reducing sedentary behavior in adolescents (Creaser et al., 2021; Danković et al., 2023). The main appeal of these devices lies in their ability to monitor physical activity levels, calories burned, and other health-related indicators (Staiano et al., 2017). However, the results of interventions targeting body composition, physical fitness, or psychological well-being remain inconclusive among overweight and obese adolescents, highlighting the need for further evidence (Staiano et al., 2017; Wang et al., 2022). This inconsistency may be partly explained by the insufficient consideration of key moderating factors, such as gender (Mateo-Orcajada et al., 2023) and baseline physical activity level (Baumann et al., 2022; Gómez-Cuesta et al., 2024).

Moreover, to date, no studies have examined wearable-based interventions promoted through physical education classes but implemented during out-of-school hours. Most previous wearable-based interventions in adolescents have been conducted outside the school context and independently of physical education curricula (Creaser et al., 2021), or have been implemented within physical education classes, where their impact is constrained by the limited instructional time allocated to physical education (Koorts et al., 2020; Chen et al., 2025). This study, which promotes the use of wearable devices through physical education classes, positioning the school as a key facilitator while simultaneously allowing adolescents to engage in physical activity autonomously outside school hours, directly addresses this research gap, making it novel compared to previous scientific literature.

Therefore, given the lack of wearable-based interventions targeting overweight and obese adolescents within the school context, particularly those promoted through physical education while extending their use beyond class time, as well as the limited understanding of how gender and baseline physical activity level may influence their effectiveness, the present study aimed to (a) determine the changes resulting from a 12-week intervention, promoted through physical education classes and involving the use of wearables during out-of-school hours, on physical activity, body composition, physical fitness, and psychological well-being of overweight or obese adolescents and (b) analyze the differences in the intervention’s impact according to the adolescents’ gender and baseline physical activity level.

Based on findings from previous interventions that employed other technological tools with overweight and obese adolescents, the following research hypotheses are proposed: H1) that the 12-week wearable-based intervention will increase physical activity levels and lead to improvements in body composition, physical fitness, and psychological well-being among overweight and obese adolescents and H2) that the intervention will be more effective in female adolescents and in those who are physically inactive at the start of the intervention.

## 2. Material and methods

### 2.1. Design

A 12-week randomized controlled trial was conducted in a secondary education school in the Region of Murcia, Spain. A non-probability convenience sample was used, which, although it limits the generalizability of the results to other populations, was necessary to ensure that participants met the inclusion criterion of being overweight or obese. This approach facilitated the recruitment of a sufficient number of adolescents within the school setting.

First, the school’s administrative team was contacted, and once approval was obtained, data collection was coordinated with the head of the physical education department. A meeting was then held with the parents and secondary school students who expressed interest in participating, during which the objectives and procedures of the study were explained, and any questions were addressed. Adolescents who remained interested in participating after the meeting provided written informed consent, signed both by themselves and their parents.

Prior to the start of the study, the institutional ethics committee of the Catholic University of Murcia (code: CE022102) approved the study design and procedures, following the guidelines of the Declaration of Helsinki and the World Medical Association. The study protocol was registered at ClinicalTrials.gov (code: NCT06140225) before the intervention began. The CONSORT guidelines were followed to structure the study design.

### 2.2. Outcomes

The primary outcomes of the study are physical activity and body composition (body mass, body mass index [BMI], waist girth, hip girth, corrected girths, fat mass, muscle mass, and sum of 3 skinfolds). These variables were selected as primary outcomes because they directly reflect the main objectives of the wearable-based intervention: increasing activity levels and improving health-related physical markers in overweight and obese adolescents.

Secondary outcomes included physical fitness (cardiorespiratory fitness, upper and lower body strength, abdominal endurance, and sprint performance) and psychological variables (life satisfaction and satisfaction of basic psychological needs). These outcomes were considered secondary because, while relevant to overall adolescent health, they are expected to be influenced indirectly by the intervention and may depend on factors such as adherence, baseline characteristics, or intervention duration.

### 2.3. Participants

**Figure 1.**
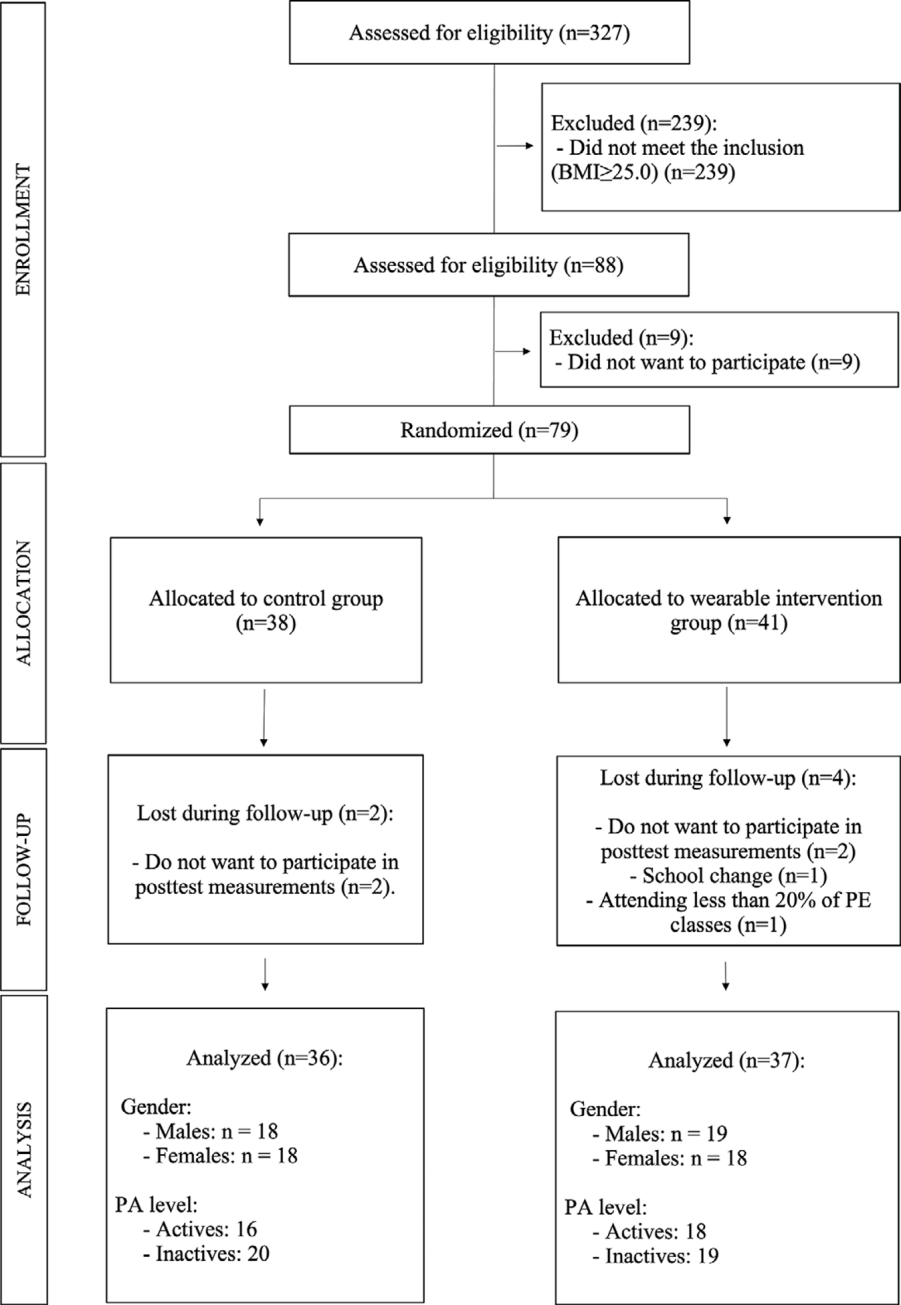
Consort flow diagram of study recruitment, attrition, and completion.

A total of 79 adolescents (mean age: 13.48 ± 1.14 years), who were all overweight or obese (mean BMI: 27.86 ± 2.47 kg/m^2^), initially participated in the study. The inclusion criteria were (a) BMI greater than 24.9 kg/m^2^; (b) age between 12 and 16 years; (c) absence of any other disease or condition associated or not associated with being overweight or obesity; and (d) enrollment in one of the four grades of compulsory secondary education. The exclusion criteria included (a) failure to complete all pre-test or post-test measurements; (b) changing schools during the study period; (c) missing more than 20% of the physical education classes; and (d) beginning or ceasing regular physical activity, such as gym training or sports practice, that could increase physical activity levels independently of the intervention. After applying these criteria, the final sample consisted of 73 adolescents (mean age: 13.44 ± 1.12 years; mean BMI: 27.73 ± 2.51 kg/m^2^) who participated voluntarily. Figure 1 presents the flow diagram of the study sample.

The sample size calculation was based on the primary outcome, perceived physical activity level (PAQ-A). Using the standard deviation reported in previous studies with adolescents using wearable devices (SD: 8.65) (Knox et al., 2019), the required sample size was estimated to detect a difference of 3.39 points with 95% confidence (*α* = 0.05) and 80% power (1−*β* = 0.80), assuming a two-tailed independent samples t-test (Serdar, Cihan, Yücel, & Serdar, 2021). This calculation indicated that a minimum of 25 adolescents per group was necessary. The calculation was performed using RStudio version 3.15.0 (RStudio Inc., USA).

### 2.4. Randomization and blinding

Adolescents were randomly assigned to either the experimental group (EG) or the control group (CG). To ensure allocation concealment, one of the researchers used a computer-generated random number table in the presence of other researchers not involved in the study. Adolescents belonging to the same class were assigned together to either the EG or CG, which led to slight differences in the initial sample size of each group. This cluster randomization was chosen to reduce a potential bias due to differences in physical activity practices during physical education classes across different groups. Randomization was not stratified by gender, as there was a similar number of males and females in each class group.

### 2.5. Instruments

#### 2.5.1. Questionnaires

Perceived physical activity was assessed using the Spanish version of the Physical Activity Questionnaire for Adolescents (PAQ-A), a validated instrument with good reliability (ICC = 0.71) (Kowalski et al., 2004; Martínez-Gómez et al., 2009). The final score of the questionnaire allows classifying adolescents as active (>2.75) or inactive (≤2.75) (Benítez-Porres et al., 2016). Life satisfaction was measured with the Spanish version of the Satisfaction with Life Scale (SWLS, *α* = 0.84) (Diener et al., 1985; Atienza et al., 2000). The satisfaction of basic psychological needs (competence, autonomy, relatedness) was assessed with the Basic Psychological Needs Satisfaction (BPNS) scale, which has demonstrated good internal consistency (*α* = 0.69–0.80). Higher scores in each instrument indicate higher levels of the construct (Wilson et al., 2006; Moreno-Murcia et al., 2011). A complete description of the instruments used can be found in Supplementary Material 1.

#### 2.5.2. Kinanthropometric and body composition measurement

Two basic variables (body mass and height), 3 skinfolds (triceps, thigh, and calf), and five girths (relaxed arm, waist, hips, thigh, and calf) were measured following the protocol of the International Society for the Advancement of Kinanthropometry (Esparza-Ros and Vaquero-Cristóbal, 2023; Esparza-Ros and Vaquero-Cristóbal, 2025), with level 3 and 4 anthropometrists performing the assessments. A complete description of the protocol and materials used for the assessment can be found in Supplementary Material 1.

Intra- and inter-rater error was calculated for the measurements taken from a subsample. Intra-rater error was 0.02% for basic measurements, 1.21% for skinfolds, and 0.04% for girths; inter-rater error was 0.03% for basic measurements, 1.98% for skinfolds, and 0.06% for girths.

Based on the anthropometric measurements, the following derived variables were calculated: BMI (body mass (kg)/height (m)^2^), muscle mass (Poortmans et al., 2005), fat mass (Slaughter et al., 1988), Σ3 skinfolds (triceps, thigh and calf), corrected girth of the arm [arm relaxed girth – (π * triceps skinfold)], thigh [middle thigh girth – (π * thigh skinfold)], and calf [calf girth – (π * calf skinfold)].

#### 2.5.3. Physical fitness test

Cardiorespiratory fitness was assessed using the 20-m shuttle run test (Léger et al., 1988; Tomkinson et al., 2019). This test allows estimating maximal oxygen consumption (VO₂ max.) using the last completed stage and Léger’s formula (Léger et al., 1988). Upper body strength was assessed using two tests: handgrip strength (España-Romero et al., 2010) and push-ups (Castro-Piñero et al., 2010). Lower body power was assessed using the countermovement jump (CMJ) test. This test involves performing a vertical jump as high as possible (Barker et al., 2018). Abdominal muscle endurance was assessed using the curl-up test (Garcia-Pastor et al., 2016). Maximal sprint speed was assessed using a 20-meter sprint test (Altmann et al., 2017; Bastida Castillo et al., 2017). A complete description of the protocol and materials used for each test can be found in Supplementary Material 1.

### 2.6. Procedure

The same protocol was applied at pre- and post-test. Participants first completed the PAQ-A, KIDMED, SWLS, and BPNS questionnaires, followed by anthropometric and body composition measurements. After a brief warm-up, adolescents performed a familiarization session for the physical fitness tests (handgrip strength, CMJ, 20-m sprint, curl-up, and push-up) and then completed the assessments according to established guidelines. The best performance of two trials was used for analysis, except for the 20-m shuttle run, which was performed once to exhaustion. This protocol has been previously used in adolescent populations (Coburn and Malek, 2014; Mateo-Orcajada et al., 2025). Full details on test execution and order are provided in Supplementary Material 1.

### 2.7. Wearables intervention

The EG initially consisted of 41 adolescents who used the Xiaomi Smart Band (Xiaomi, Beijing, China) for a period of 12 weeks. A total of 37 adolescents completed the intervention. The CG did not use any wearable device during the 12-week period, and both groups were asked to continue their regular physical and sports activities as usual. Both the EG and the CG continued attending their physical education classes as normal.

The Xiaomi Smart Band was selected because it has been used in previous studies aimed at promoting physical activity in adolescents and has been found to be user-friendly for this population (Mayorga-Vega et al., 2022). Furthermore, it demonstrates a good criterion validity and shows a moderate to high accuracy in step counting when compared with ActiGraph accelerometers (Casado-Robles et al., 2023; Alvarenga et al., 2025).

During the 12-week intervention, the use of wearables was promoted during physical education classes. Adolescents were instructed to wear the device daily, but only sessions specifically recorded on the device counted toward meeting intervention objectives. Participants were asked to record at least three training sessions per week. Other activities and daily steps were automatically logged by the device but were not considered for evaluating adherence to weekly step goals.

The session-specific step goals started at a minimum of 5,000 steps in the first week, as values below this threshold are associated with a sedentary lifestyle (C Tudor-Locke et al., 2004; Morency et al., 2007), and gradually increased by 600 steps per week to reach between 12,000 and 13,000 steps per session in the final week. These targets were set to reflect sufficient activity to consider adolescents active or highly active and improve cardiorespiratory fitness and health (Epstein et al., 2001; Tudor-Locke and Bassett, 2004; Mateo-Orcajada et al., 2025).

Adherence to the intervention was monitored weekly using the device logs, and a researcher tracked which adolescents completed or missed their weekly targets, as well as any dropouts. The EG participants who completed the entire intervention received a bonus in their final physical education grade, following the methodology adopted in previous school-based interventions to enhance adherence (Hardman et al., 2011). Adolescents who did not meet the weekly step goal in a given week were not excluded, as they could meet the target in subsequent weeks. In this way, participants could continue and complete as much of the intervention as possible at their own pace.

The full intervention completion rate was 32.43% (12/37). The attrition rate for the EG was 9.76% (4/41). The reasons for dropping out were a) refusal to participate in the post-test measurements (*n* = 2), b) changing school (*n* = 1), and c) attending less than 20% of the PE classes (*n* = 1). Only when adolescents completely stopped using the wearable device was this considered withdrawal from the intervention. Some participants did not meet the weekly step goal established for certain weeks; however, this was not considered abandonment, as they continued to wear the device and record physical activity sessions. Table 1 summarizes the weekly step goals, the percentage of adolescents who met each weekly target, and the percentage who continued to use the wearable device despite not achieving the prescribed goal.

**Table 1.** Weekly adherence to the wearable-based intervention: step goals, compliance, and continued device use

Week	Steps per session	Weekly volume (steps)	Adolescents who continue in the intervention (%)	Adolescents who complete the weekly goal (%)
1	5,000	15,000	100	100
2	5,684	17,051	97.30	97.30
3	6,367	19,102	97.30	91.89
4	7,051	21,153	94.59	89.19
5	7,735	23,204	89.19	81.08
6	8,418	25,255	89.19	75.68
7	9,102	27,305	89.19	67.57
8	9,785	29,356	89.19	59.46
9	10,469	31,407	89.19	56.76
10	11,153	33,458	89.19	51.35
11	11,836	35,509	89.19	43.24
12	12,520	37,560	89.19	32.43

Based on previous scientific evidence suggesting that technology-based physical activity interventions lasting at least 8 weeks are more likely to induce measurable changes in adolescents (He et al., 2021; Martínez-Gómez et al., 2009), a sensitivity analysis was conducted according to intervention exposure. Participants were classified into a short intervention exposure group (≤7 weeks of participation) (*n* = 15) and a long intervention exposure group (≥8 weeks of participation) (*n* = 22), based on the number of weeks they were able to meet the weekly target set.

### 2.8. Statistical analysis

The normality of the data was analyzed using the Kolmogorov–Smirnov test, as well as a skewness and kurtosis analysis. Since the sample followed a normal distribution, parametric tests were used for the analysis. The mean (M) and standard deviation (SD) were used as descriptive statistics of the sample. Baseline differences between the EG and CG were examined using independent samples t-tests to ensure group comparability prior to the intervention. To examine the effects of the intervention over time, an ANOVA was conducted for all dependent variables. Additionally, repeated-measures analyses of covariance were performed to assess the influence of gender and baseline physical activity level as covariates. To further explore subgroup effects, two multivariate analysis of variance (MANOVAs) were conducted separately for the EG and CG, considering gender (male versus female) and baseline physical activity level (active versus inactive). Between-group differences in intervention effects were analyzed using ANOVA on change scores, calculated as the difference between post- and pre-intervention values (Δ = post − pre), comparing changes in the EG and CG. In addition, exploratory analyses within the EG were conducted according to the degree of exposure to the intervention, in order to examine the influence of adherence on the observed outcomes. Post hoc comparisons were adjusted using the Bonferroni correction to control for multiple testing. Partial eta squared (*η*^2^) was used to calculate effect size, following conventional benchmarks: small (*η*^2^ ≥ 0.01), medium (*η*^2^ ≥ 0.06), and large (*η*^2^ ≥ 0.14). A value of *p* < 0.05 was set to determine statistical significance. The statistical analysis was performed with the SPSS statistical package (v.25.0; SPSS Inc., IL).

## 3. Results

Table S1 (Supplementary Material 2) contains the baseline values for the CG and EG in the study variables. No significant differences were found at the start of the study in any of the variables analyzed between the two groups (*p* > 0.106).

### 3.1. Main intervention effects (primary and secondary outcomes)

Regarding the primary outcomes, adolescents in the EG showed a significant increase in perceived physical activity following the intervention (*p* = 0.048), corresponding to a mean absolute increase of 0.19 points on the PAQ-A scale (relative change: 7.31%) and a moderate effect size (*η*^2^ = 0.09). In contrast, no significant changes in perceived physical activity were observed in the CG (*p* > 0.05) (Table 2). However, no significant differences were found in the change between pre- and post-test in the CG and EG for this variable (*p* = 0.141) (Table 3).

**Table 2.** Differences in the study variables between the pre- and post-test in the experimental and control groups

Variable	Group	Timepoint							Wearable use * gender			Wearable use * PA level		
		Pre	Post	Pre–post diff.	F	*p*	95% CI diff.	*η*^2^	F	*p*	95% CI diff.	F	*p*	95% CI diff.
Primary outcomes														
Physical activity	CG	2.69 ± 0.68	2.70 ± 0.61	−0.01 ± 0.09	0.01	0.920	−0.20; 0.18	0.00	0.39	0.538	−0.24; 0.13	0.04	0.846	−0.19;0.16
	EG	2.61 ± 0.70	2.80 ± 0.62	−0.19 ± 0.09	4.16	0.048	−0.38; −0.00	0.09	2.66	0.110	−0.33; 0.04	4.42	0.041	−0.36; −0.01
Body Mass (kg)	CG	71.93 ± 10.50	73.07 ± 11.70	−1.15 ± 0.55	4.27	0.045	−2.26; −0.03	0.10	2.57	0.117	−2.02;0.23	4.17	0.048	−2.28; −0.01
	EG	72.51 ± 10.78	72.47 ± 11.28	0.05 ± 0.50	0.01	0.928	−0.97;1.06	0.00	0.10	0.752	−1.18;0.86	0.01	0.928	−0.99;1.08
BMI (kg/m^2^)	CG	27.99 ± 2.44	28.07 ± 2.80	−0.08 ± 0.21	0.13	0.720	−0.50;0.35	0.00	0.04	0.844	−0.49;0.40	0.14	0.714	−0.51;0.35
	EG	27.78 ± 2.60	27.23 ± 2.56	0.55 ± 0.19	8.14	0.007	0.16;0.94	0.17	6.85	0.013	0.12;0.93	8.03	0.007	0.16;0.94
Hip girth (cm)	CG	101.46 ± 6.00	102.75 ± 6.39	−1.30 ± 0.51	6.40	0.016	−2.33; −0.26	0.14	4.94	0.072	−2.24; −0.11	6.18	0.017	−2.34; −0.24
	EG	102.70 ± 5.12	102.19 ± 5.16	0.50 ± 0.45	1.23	0.274	−0.41; 1.42	0.03	0.77	0.385	−0.53;1.35	1.18	0.284	−0.43;1.43
Fat mass (%)	CG	35.11 ± 5.70	34.62 ± 6.13	0.49 ± 0.32	2.36	0.131	−0.15;1.14	0.05	1.73	0.195	−0.23;1.09	2.30	0.136	−0.16;1.14
	EG	34.90 ± 5.08	32.95 ± 4.57	1.96 ± 0.33	35.86	<0.001	1.30;2.62	0.44	36.65	<0.001	1,35;2.69	35.62	<0.001	1.30;2.62
Sum of 3 skinfolds	CG	89.83 ± 28.00	91.53 ± 27.48	−1.70 ± 1.99	0.73	0.398	−5.71;2.31	0.02	0.25	0.620	−5.02;3.02	0.72	0.402	−5.76;2.35
	EG	95.14 ± 22.35	88.76 ± 20.94	6.38 ± 1.94	10.78	0.002	2.46;10.30	0.20	8.62	0.005	1.79;9.64	10.55	0.002	2.42;10.35
Secondary outcomes														
Curl-up (reps)	CG	16.92 ± 11.68	20.79 ± 13.09	−3.88 ± 1.67	5.48	0.024	−7.21; −0.54	0.11	5.36	0.025	−7.39; −0.51	5.36	0.025	−7.25; −0.50
	EG	14.13 ± 8.93	19.74 ± 7.57	−5.61 ± 1.69	11.01	0.002	−9.01; −2.20	0.20	10.05	0.003	−9.05; −2.01	10.76	0.002	−9.06; −2.16

Abbreviation: BMI, body mass index.

**Table 3.** Changes between the experimental and control groups after the intervention in the study variables

Variable	Wearable use				
	Pre–post CG – Pre–post EG	F	*p*	95% CI diff.	*η*^2^
Primary outcomes					
Physical activity	0.24 ± 0.16	2.27	0.141	−0.08; 0.56	0.06
Body mass (Kg)	−1.94 ± 0.72	7.21	0.011	−3.40; −0.47	0.17
BMI (kg/m^2^)	−0.65 ± 0.31	4.44	0.042	−1.27; −0.30	0.11
Hips girth (cm)	−2.01 ± 0.73	7.49	0.010	−3.49; −0.52	0.17
Fat mass (%)	−1.55 ± 0.56	7.58	0.009	−2.70; −0.41	0.14
Sum of 3 skinfolds	−8.58 ± 3.36	6.54	0.015	−15.39; −1.77	0.15
Secondary outcomes					
Curl-up (reps)	1.41 ± 2.21	0.41	0.528	−3.07;5.89	0.01

Abbreviation: BMI, body mass index.

For body composition, the EG showed a significant decrease in BMI (*p* = 0.007; mean diff: 0.55; relative change: 1.98%), fat mass (*p* < 0.001; mean diff: 1.96; relative change: 5.59%), and sum of 3 skinfolds (*p* = 0.002; mean diff: 6.38; relative change: 6.71%), with a large effect size for all differences analyzed. In the CG, significant differences were also found, notably an increase in body mass (*p* = 0.045; mean diff: −1.15; relative change: 1.58%) and hips girth (*p* = 0.016; mean diff: −1.30; relative change: 1.27%), with a moderate effect size (Table 2). Analysis of the pre- post-test change between the CG and EG showed significant differences in body mass (*p* = 0.011; mean diff: −1.94), BMI (*p* = 0.042; mean diff: −0.65), hips girth (*p* = 0.010; mean diff: −2.01), fat mass (*p* = 0.009; mean diff: −1.55), and sum of 3 skinfolds (*p* = 0.015; mean diff: −8.58) (Table 3).

Table 2 also shows the effects of the covariates in these variables. For the EG, gender did not have a significant influence on the physical activity level (*p* = 0.110), although it did affect BMI (*p* = 0.013), fat mass (*p* < 0.001), and sum of 3 skinfolds (*p* = 0.005). The physical activity level covariate also showed a significant influence in the EG, specifically on physical activity practiced (*p* = 0.041), BMI (*p* = 0.007), fat mass (*p* < 0.001), and sum of 3 skinfolds (*p* = 0.002), as well as in the CG, mainly affecting body mass (*p* = 0.048) and hips girth (*p* = 0.017).

According to the secondary outcomes, significant differences were observed in curl-up in both the EG (*p* = 0.002; mean diff: −5.61; relative change: 39.70%) and CG (*p* = 0.024; mean diff: −3.88; relative change: 22.87%), with an increase in the performance of this test. The effect size was large for both groups (Table 2). However, an analysis of the pre- and post-test change between the CG and EG showed no significant differences in curl-up (*p* = 0.528) (Table 3).

Both the gender covariate (EG: *p* = 0.003; CG: *p* = 0.025) and previous physical activity level (EG: *p* = 0.002; CG: *p* = 0.025) affected curl-up performance in the EG and CG (Table 2).

Table S2 (Supplementary Material 2) shows the complete results with the body composition, psychological, and physical fitness variables that did not show significant differences (*p* > 0.05), as well as the effect of the covariates on these variables. Similarly, Table S3 (Supplementary Material 2) shows the analysis of the change between the pre- and post-test in the CG and EG for all variables.

### 3.2. Analysis of differences in study variables according to intervention exposure in the experimental group

Table 4 shows the differences among adolescents in the EG according to whether their exposure to the intervention was short or long. The results revealed significant differences in the primary outcomes. Thus, in the long-exposure group, an increase in physical activity level (*p* = 0.032; mean diff: −0.33; relative change: 13.49%), as well as a decrease in fat mass (*p* = 0.015; mean diff: 4.13; relative change: 10.77%) and in the sum of 3 skinfolds (*p* < 0.001; mean diff: 11.89; relative change: 12.38%) were found. A large effect size was found for all differences. The short-exposure group showed no significant differences in these variables after the intervention (*p* > 0.247).

**Table 4.** Pre–post changes in physical activity, body composition, psychological, and physical fitness variables according to intervention exposure length

Variable	Exposure	Pre	Post	Mean diff.	*p*	95% CI diff.	*η*^2^
Primary outcomes							
Physical activity	Long	2.52 ± 0.59	2.86 ± 0.59	−0.33	0.032	−0.69; −0.03	0.25
	Short	2.65 ± 0.78	2.76 ± 0.67	−0.10	0.468	−0.39; 0.19	0.03
Fat mass (%)	Long	38.33 ± 6.26	34.20 ± 6.52	4.13	0.015	0.88; 7.38	0.25
	Short	41.33 ± 10.67	40.67 ± 8.91	0.67	0.600	−1.94; 3.27	0.01
Sum of 3 skinfolds	Long	93.67 ± 18.58	81.78 ± 17.00	11.89	<0.001	5.71; 18.07	0.43
	Short	96.08 ± 25.11	93.24 ± 22.56	2.84	0.247	−2.12; 7.80	0.06
Secondary outcomes							
Curl-up (reps)	Long	11.33 ± 7.45	19.33 ± 7.45	−8.00	0.002	−12.57; −3.43	0.39
	Short	15.93 ± 9.58	20.00 ± 7.91	−4.07	0.031	−7.73; −0.41	0.20

Regarding the secondary outcomes, both the long-exposure (*p* = 0.002; mean diff: −8.00; relative change: 70.60%) and the short-exposure (*p* = 0.031; mean diff: −4.07; relative change: 25.55%) groups showed significant improvements only in curl-up performance, with a large effect size.

The complete results with body composition, psychological, and physical fitness variables that did not show significant differences (*p* > 0.05) are provided in Table S4 (Supplementary Material 2).

### 3.3. Gender-specific effects

**Figure 2.**
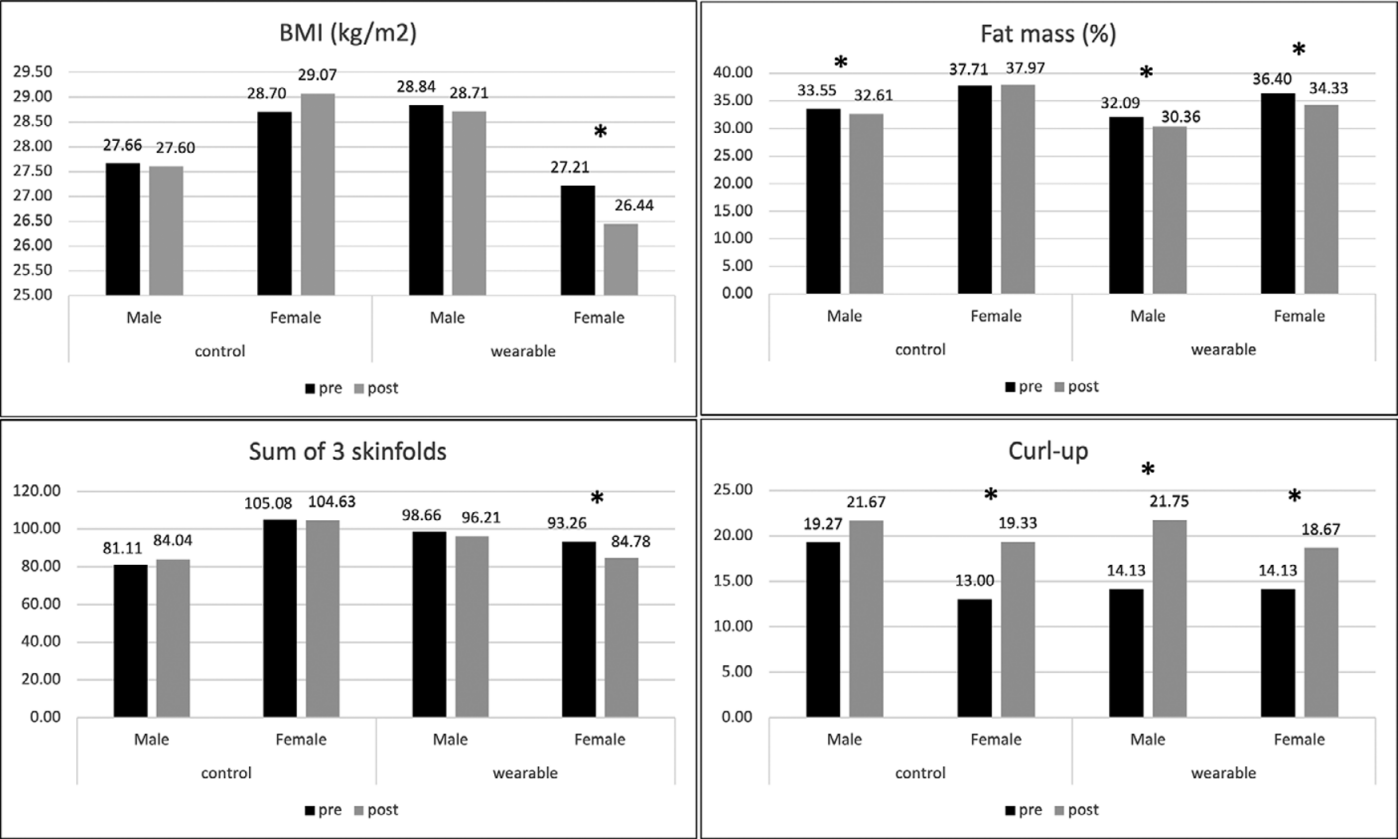
Analysis of the differences in the study variables according to gender.

Figure 2 shows the variables in which significant differences were found according to gender. For the primary outcomes, males in the EG (*p* = 0.003; mean diff: 1.73; relative change: 5.39%) and the CG (*p* = 0.022; mean diff: 0.94; relative change: 2.80%) showed significant differences only in fat mass, which decreased after the intervention period. The effect sizes for both groups were large (*η*^2^ > 0.17). However, the analysis of the change between the two groups was not significant (*p* = 0.400) (Table 5).

**Table 5.** Changes between the experimental and control groups after the intervention in the study variables according to gender

Variable	Gender	Wearable use				
		Pre–post CG – Pre–post EG	F	*p*	95% CI diff.	*η*^2^
Primary outcomes						
BMI (kg/m^2^)	Male	−0.41 ± 0.45	0.82	0.379	−1.36; 0.55	0.05
	Female	−0.82 ± 0.49	2.83	0.110	−1.84; 0.20	0.136
Fat mass (%)	Male	−0.80 ± 0.92	0.75	0.400	−2.75; 1.16	0.05
	Female	−2.74 ± 0.72	14.59	0.001	−4.24; −1.23	0.45
Sum of 3 skinfolds	Male	−6.64 ± 4.78	1.93	0.184	−16.77; 3.49	0.11
	Female	−6.69 ± 5.09	1.73	0.205	−17.39; 4.01	0.09
Secondary outcomes						
Curl-up	Male	4.23 ± 3.10	1.86	0.191	−2.34; 10.79	0.10
	Female	−1.27 ± 3.54	0.13	0.724	−8.70; 6.17	0.01

Abbreviation: BMI, body mass index.

In the case of females, those belonging to the EG showed a significant decrease in BMI (*p* = 0.002; mean diff: 0.77; relative change: 2.83%), fat mass percentage (*p* < 0.001; mean diff: 2.08; relative change: 5.71%), and sum of 3 skinfolds (*p* < 0.001; mean diff: 8.48; relative change: 8.60%), while those belonging to the CG showed no significant differences in the primary outcomes. The effect sizes for the differences in the EG were large (*η*^2^ > 0.21) (Figure 2). Regarding the change between the pre–post in the CG and EG, only significant differences were found in fat mass (*p* = 0.001; mean diff: −2.74) (Table 5).

According to the secondary outcomes, significant differences were only found in the curl-up test performance. Females in the CG (*p* = 0.024; mean diff: −6.33; relative change: 48.69%) and EG (*p* = 0.036; mean diff: −4.53; relative change: 32.06%), as well as males in the EG (*p* = 0.011; mean diff: −7.63; relative change: 53.99%) showed a significant increase in this test (Figure 2). The effect sizes were large for all the differences (*η*^2^ > 0.20). However, an analysis of the pre- and post-test change between the CG and EG showed no significant differences in curl-up (males: *p* = 0.191; females: *p* = 0.724) (Table 5).

### 3.4. Intervention effects according to baseline physical activity level

**Figure 3.**
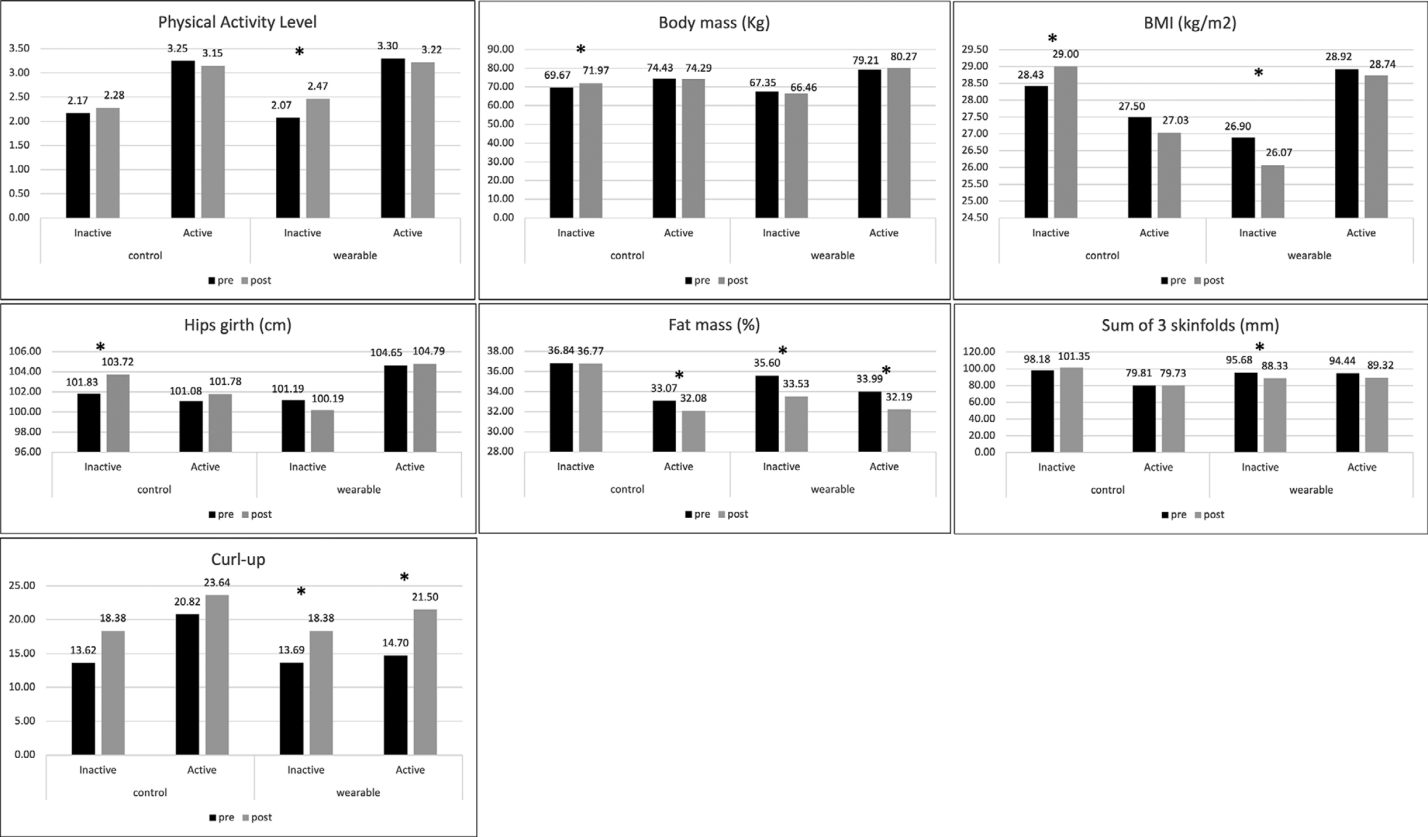
Analysis of the differences in the study variables according to the previous level of physical activity.

The differences based on the adolescents’ physical activity level prior to the start of the intervention are shown in Figure 3. For the primary outcomes, inactive adolescents from the CG showed an increase in body mass (*p* = 0.002; mean diff: −2.30; relative change: 3.30%), BMI (*p* = 0.037; mean diff: −0.57; relative change: 2.00%), and hips girth (*p* = 0.012; mean diff: −1.89; relative change: 1.86%); meanwhile, inactive adolescents from the EG showed a significant increase in physical activity level (*p* = 0.001; mean diff: −0.40; relative change: 19.32%), along with a decrease in BMI (*p* < 0.001; mean diff: 0.83; relative change: 3.09%), fat mass percentage (*p* < 0.001; mean diff: 2.07; relative change: 5.81%), and the sum of 3 skinfolds (*p* = 0.008; mean diff: 7.35; relative change: 7.68%). The effect sizes were large for all the differences found (*η*^2^ > 0.17). The analysis of the change between the two groups showed significant differences in body mass (*p* < 0.001; mean diff: −3.72), BMI (*p* = 0.008; mean diff: −1.21), hip girth (*p* = 0.008; mean diff: −2.93), fat mass (*p* = 0.011; mean diff: −2.23), and the sum of 3 skinfolds (*p* = 0.049; mean diff: −11.45) (Table 6).

**Table 6.** Changes between the experimental and control groups after the intervention in the study variables according to the previous level of physical activity

Variable	Gender	Wearable use				
		Pre-–post CG – Pre–post EG	F	*p*	95% CI diff.	*η*^2^
Primary outcomes						
Physical activity level	Inactive	0.37 ± 0.26	2.09	0.165	−0.17; 0.90	0.10
	Active	0.06 ± 0.11	0.29	0.601	−0.18; 0.30	0.02
Body mass (kg)	Inactive	−3.72 ± 0.81	21.33	<0.001	−5.41; −2.04	0.53
	Active	0.23 ± 1.09	0.05	0.832	−2.08; 2.55	0.00
BMI (kg/m^2^)	Inactive	−1.21 ± 0.41	8.82	0.008	−2.07; −0.36	0.32
	Active	0.02 ± 0.43	0.00	0.973	−0.90; 0.93	0.00
Hip girth (cm)	Inactive	−2.93 ± 0.98	8.92	0.008	−4.98; −0.88	0.32
	Active	−0.88 ± 1.10	0.63	0.438	−3.22; 1.47	0.04
Fat mass (%)	Inactive	−2.23 ± 0.79	7.96	0.011	−3.88; −0.58	0.30
	Active	−0.76 ± 0.80	0.91	0.356	−2.47; 0.94	0.06
Sum of 3 skinfolds	Inactive	−11.45 ± 5.45	4.42	0.049	−22.86; −0.049	0.19
	Active	−5.15 ± 3.60	2.05	0.173	−12.83; 2.53	0.12
Secondary outcomes						
Curl-up	Inactive	1.32 ± 2.73	0.23	0.634	−7.02; 4.39	0.01
	Active	1.66 ± 3.73	0.20	0.663	−6.30; 9.61	0.01

Abbreviation: BMI, body mass index.

For active adolescents, those belonging to the CG (*p* = 0.042; mean diff: 0.99; relative change: 2.99%) and the EG (*p* < 0.001; mean diff: 1.81; relative change: 5.33%) showed a significant decrease in fat mass percentage. The effect sizes were large for both groups (*η*^2^ > 0.14) (Figure 3). However, no significant differences were observed when analyzing the change between pre- and post-test in both groups for this variable (*p* = 0.356) (Table 6).

According to the secondary outcomes, both active (*p* = 0.012; mean diff: −6.80; relative change: 46.26%) and inactive (*p* = 0.046; mean diff: −4.69; relative change: 34.26%) adolescents from the EG showed a significant increase in the curl-up test performance. The effect size was large for both groups (*η*^2^ > 0.19) (Figure 3). However, no significant differences were observed when compared with the change between pre- and post-test in both inactive and active groups for this variable (inactive: *p* = 0.634; active: *p* = 0.663) (Table 6).

## 4. Discussion

The present study examined the effects of a 12-week wearable-based intervention promoted through physical education classes on primary outcomes (physical activity and body composition) and secondary outcomes (physical fitness and psychological well-being) in overweight or obese adolescents. The main findings indicate that the intervention produced clearer and more consistent effects on the primary outcomes, particularly on body composition, whereas effects on secondary outcomes were limited or absent. Specifically, the intervention was associated with modest increases in perceived physical activity and meaningful improvements in body composition, particularly in fat-related variables, whereas effects on physical fitness were limited and no significant changes were observed in psychological outcomes. Importantly, the benefits of the intervention were not uniform across the sample, being more pronounced among females, previously inactive adolescents, and those with longer exposure to the intervention, while some improvements, such as gains in abdominal endurance, were also observed in the CG. These results highlight both the potential and the limitations of wearable-based interventions in school settings and underscore the importance of adherence, baseline characteristics, and intervention design when interpreting their effectiveness.

### 4.1. Main intervention effects (primary and secondary outcomes)

The first objective of the present study was to determine the changes produced by a 12-week intervention promoted through physical education classes, during which wearables were used outside school hours, on physical activity, body composition, physical fitness, and psychological well-being of overweight or obese adolescents. Regarding the primary outcomes, a significant increase in perceived physical activity was observed in the EG, whereas no changes were found in the CG. However, the magnitude of this increase was not sufficient to yield statistically significant differences when compared with the CG, suggesting a modest effect of the intervention on perceived physical activity. This finding contrasts with previous studies reporting declines in physical activity following wearable use (Kerner et al., 2019). This may be explained by evidence indicating that wearables primarily increase daily step counts but do not appear to affect moderate-to-vigorous physical activity as much (Au et al., 2024). Given this, in populations with very low baseline activity levels, such as overweight or obese adolescents (Olds et al., 2011), increases in daily steps may nevertheless be perceived as meaningful improvements in overall physical activity.

In contrast, the intervention showed more robust effects on body composition, which constituted a key primary outcome of the study. Adolescents in the EG exhibited significant reductions in BMI, fat mass, and the sum of 3 skinfolds, whereas the CG showed increased body mass and hip girth. Importantly, the pre–post changes in body mass, BMI, hip girth, fat mass, and sum of 3 skinfolds differed significantly between groups, indicating that the wearable-based intervention could have a protective or beneficial effect on fat-related body composition variables. These findings are consistent with previous evidence showing that physical activity intervention programs, typically lasting 12 to 14 weeks and increasing daily steps, are particularly effective in improving body fat indicators in adolescents (Soares et al., 2023; Stojanović et al., 2024). In fact, these benefits have been observed mainly in overweight or obese adolescents who tend to show lower levels of physical activity as compared to their normal-weight peers (Olds et al., 2011; Park et al., 2018). The relevance of these findings lies in the fact that interventions involving wearables could become a useful tool to achieve changes in body composition, particularly in fat-related variables, among adolescents who are overweight or obese, an essential consideration given the health risks associated with excessive fat accumulation in this population (Kelly et al., 2024). However, future studies are needed to analyze the potential of wearables in this field.

Regarding the secondary outcomes, the effects of the intervention were more limited. Improvements were observed only in abdominal muscular endurance (curl-up test), and these gains occurred in both the EG and CG. This suggests that factors unrelated to the wearable intervention, such as growth, maturation, or exposure to regular physical education classes, may have contributed to the observed improvements, as has been observed in previous research (Lourenço, Freitas, & Rakaa, 2025; Martínez-Romero et al., 2025). This does not mean that the proposed program with wearables had no effect on curl-up performance, as the relative change was greater in the EG than in the CG. Walking, when performed regularly and systematically, has been shown to be sufficient to improve abdominal muscular endurance, due to the stabilization demands it entails (Shnayderman and Katz-Leurer, 2013). However, the fact that the CG also showed significant differences and that there were no differences between the change in the EG and CG suggests that other factors are involved in these differences, with further research needed to indicate that programs based on increasing daily step counts using wearables are useful in this regard.

A surprising result of the present study was that there were no differences in the cardiorespiratory capacity of adolescents, which is contrary to other previous research with technological devices (Mateo-Orcajada et al., 2024). The differential effects observed between body composition and physical fitness outcomes may be explained by the nature of the physical activity promoted through the wearable-based intervention. The intervention primarily encouraged increases in daily step count, which likely led to higher overall energy expenditure and, consequently, reductions in fat-related variables (Stojanović et al., 2024). However, improvements in cardiorespiratory fitness typically require sustained engagement in moderate-to-vigorous physical activity at sufficient intensity and duration, which may not have been achieved through step-based goals alone (Landgraff et al., 2021). Therefore, the wearable intervention may have been effective in promoting behavioral changes related to movement volume, but not intensity, which could explain the observed improvements in body fat without corresponding gains in cardiorespiratory fitness and other physical fitness variables.

Furthermore, no significant changes were detected in any psychological variables, indicating that the intervention, as implemented, was insufficient to produce short-term improvements in psychological well-being. These findings differ from those of previous research, which showed that mobile apps-based PA interventions have beneficial effects on adolescents’ psychological well-being by enhancing competence, autonomy, relatedness, or intrinsic motivation (Cowley et al., 2024; Mateo-Orcajada et al., 2024b). The absence of changes in these variables could be due to the design of the intervention. Each adolescent had to record their workouts on their wearable device, with only those involving walking being valid, and no other alternative activities were offered to help them achieve their goal. Furthermore, no cooperative challenges or interactions were created to help motivate the adolescents, despite this being a highly relevant aspect indicated in previous research (Marker & Staiano, 2015), and one that should be considered in future intervention designs involving wearables.

### 4.2. Analysis of differences in study variables according to intervention exposure in the experimental group

A key contribution of the present study is the analysis of outcomes according to the degree of exposure to the intervention of the adolescents in the EG, which directly addresses the issue of adherence. Although only a minority of participants completed the full 12-week intervention, a substantial proportion of adolescents maintained partial engagement over several weeks. The results showed that adolescents with longer exposure to the intervention experienced significant improvements in primary outcomes, including increases in physical activity and reductions in fat mass and sum of 3 skinfolds. In contrast, adolescents with shorter exposure showed no significant changes in these variables. These findings suggest that sustained engagement with the wearable intervention is a critical determinant of its effectiveness, particularly for body composition outcomes. These results are consistent with those of previous interventions using different training programs (high intensity, mobile app-based), which showed that the greater the adherence and degree of completion, the greater the changes observed, mainly in variables related to fat mass (de Freitas, Zago, Antônio, Brandão, & Videira-Silva, 2025; Domaradzki, Koźlenia, & Popowczak, 2023; Mateo-Orcajada et al., 2024a).

Interestingly, improvements in abdominal muscular endurance were observed in both short- and long-exposure groups, indicating that some benefits may be achieved even with limited exposure. However, the absence of changes in primary outcomes among short-exposure participants highlights the importance of adherence and supports the interpretation that the most meaningful health benefits require a minimum duration of engagement. These findings are especially relevant given the well-documented challenges of adherence in interventions targeting overweight or obese adolescents (Guijo et al., 2024; Sundar, Løndal, Lagerløv, Glavin, & Helseth, 2018). Rather than excluding partially adherent participants, the present approach offers a more valid representation from the perspective of real-world implementation, where intermittent participation is common. Nevertheless, the low full-completion rate remains a critical limitation and should be considered when interpreting the overall effectiveness of the intervention.

Based on the results obtained, the first research hypothesis (H1), which stated that a 12-week intervention using wearables would increase physical activity levels and lead to improvements in body composition, physical fitness, and psychological well-being among overweight and obese adolescents, can be partially accepted. The intervention produced more consistent effects on the primary outcomes, particularly on body composition, with significant reductions in BMI, fat mass, and the sum of 3 skinfolds observed in the EG and significant differences compared with the CG. In addition, a modest increase in perceived physical activity was found in the EG, although this change was not large enough to result in significant differences compared to the CG. In contrast, the effects on secondary outcomes were limited. Improvements in abdominal muscular endurance were observed in both the EG and CG, suggesting that these gains may not be exclusively attributable to the wearable-based intervention. Furthermore, no significant changes were found in any psychological well-being variables, indicating that the intervention, as designed, was insufficient to produce short-term psychological benefits. These findings suggest that the intervention was more effective in improving fat-related body composition outcomes, while its impact on physical fitness and psychological well-being was limited, underscoring the importance of intervention design, adherence, and outcome selection when evaluating wearable-based programs in school settings.

### 4.3. Gender-specific effects

The second objective of the study was to analyze the differences in the impact of the intervention according to gender and the adolescents’ prior physical activity level. Gender-specific analyses revealed that the intervention effects on primary outcomes were more pronounced among females. While both males and females in the EG showed reductions in fat mass, only females exhibited significant decreases in BMI and the sum of 3 skinfolds. Moreover, the only significant between-group difference observed after the intervention was the reduction in fat mass among females. These findings are particularly relevant given that adolescent females, especially those who are overweight or obese, tend to engage in lower levels of physical activity and derive fewer benefits from traditional interventions, as their increase in daily physical activity and daily minutes of physical activity is lower (Goldfield et al., 2008; Sejdija and Maggio, 2025). The present results suggest that wearable-based interventions may help mitigate these disparities by providing an accessible form of physical activity. While technological resources such as mobile applications have not shown a great effectiveness in producing changes in body composition, particularly in fat-related variables in adolescents who are overweight and obese (Mateo-Orcajada et al., 2024), wearables appear to be effective for this purpose, mainly for females. Therefore, promoting these types of tools in physical education classes could be considered in order to maximize their benefits.

Regarding secondary outcomes, improvements in curl-up performance were observed in females from both groups and in males from the EG. However, the analysis of change between the EG and CG was not significant for neither males nor females. It is important to highlight that adolescent males tend to have higher physical activity levels and greater abdominal strength and endurance (De Marco et al., 2025), which could explain why no changes in curl-up performance were observed in males from the CG, since there was no stimulus for improvement. In contrast, females in the CG did show a significant increase in performance, which could be because they generally have lower strength and endurance in this musculature (De Marco et al., 2025), so the stimulus generated by physical education classes alone might be sufficient to produce significant changes in this test, as shown in previous studies (Lasković et al., 2022; Batista Lemes et al., 2023). Regarding males and females in the EG, previous studies have shown that physical activity is effective in increasing abdominal muscle strength and endurance in adolescents (Schaeffer et al., 2024). It was observed that adolescents who practice regular physical activity showed greater abdominal strength and endurance than those who simply maintained an active lifestyle (Schaeffer et al., 2024). Therefore, the increase in daily steps and physical activity level observed in adolescents from the EG could be responsible for the improvement in curl-up performance. However, this statement should be viewed with caution, since there were no differences when analyzing the change between the pre- and post-tests in this variable when comparing the CG and EG. This lends greater consistency to the interpretation that these changes may be influenced by maturation or general exposure to physical education, rather than by the intervention itself.

With regard to psychological outcomes, no significant improvements were observed in either males or females following the intervention, nor were there significant differences between the EG and CG. These findings suggest that the wearable-based intervention, as implemented, was insufficient to elicit short-term changes in psychological well-being, regardless of gender. These results are contrary to those observed in previous studies, which found gender differences in the benefits obtained after physical activity interventions, with males receiving greater benefits (Fu, Li, Li, & Wang, 2025). The absence of gender-specific psychological effects in the present study may therefore be explained by the relatively simple structure of the intervention, which focused primarily on step accumulation without explicitly targeting psychological constructs. These findings highlight the need for future wearable-based interventions to integrate motivational and psychosocial components if improvements in psychological well-being are to be achieved in adolescents, particularly in populations at risk, such as those who are overweight or obese.

### 4.4. Intervention effects according to baseline physical activity level

Baseline physical activity level also moderated the intervention effects. Among inactive adolescents, those in the EG showed significant improvements in multiple primary outcomes, including physical activity, BMI, fat mass, and the sum of 3 skinfolds, whereas inactive adolescents in the CG exhibited unfavorable changes. In contrast, among active adolescents, the intervention effects were more limited and restricted to reductions in fat mass. These results are consistent with findings from previous studies that used physical activity mobile applications in active and inactive normal-weight adolescents, where decreases in variables related to body fat were observed in both groups, regardless of prior physical activity level (Gómez-Cuesta et al., 2024). The benefits of regular physical activity practice, regardless of health status or prior activity level, are well established (Granger et al., 2017; Tambalis, 2022) and also demonstrated in the present study, as both active and inactive adolescents who participated in the wearable intervention experienced benefits. However, the fact that inactive, overweight, and obese adolescents showed significant improvements in body composition, mainly in fat-related variables, after using wearables, is highly relevant as it provides scientific evidence in an area where clear conclusions have not been established (Staiano et al., 2017; Wang et al., 2022). Thus, wearables could be a tool with a great potential when incorporated and promoted through physical education classes to produce changes in inactive adolescents who are overweight and obese, as they offer them an alternative to traditional sports or other forms of activity that they may be less attracted to due to fear of being judged (Skogen et al., 2022).

Therefore, one of the most important aspects of the present study was the finding that the changes between the CG and the EG after the intervention were significant only in the group of inactive adolescents. This is highly important, as this population of overweight and obese adolescents is considered a hard-to-reach population, being quite reluctant to physical activity interventions, and previous research results have not been very promising, even in studies that included technological and digital tools (Vajravelu and Arslanian, 2021). In view of this results, this study provides an initial scientific approximation that a 12-week intervention with wearable devices, promoted through physical education classes, could be particularly effective for adolescents with lower baseline activity levels, who also represent a group at greater health risk and are typically harder to engage in structured physical activity programs. However, further research is needed to corroborate these results and establish accurate conclusions.

Regarding secondary outcomes, the effects of the intervention differed according to the adolescents’ baseline physical activity level, although these effects were limited in scope. Improvements in abdominal muscular endurance were observed only among adolescents in the EG, regardless of whether they were classified as active or inactive at baseline, whereas no significant changes were detected in the CG. This suggests that the wearable-based intervention may have contributed to gains in this specific fitness component. Nevertheless, the absence of significant between-group differences indicates that these improvements should be interpreted with caution and may also reflect maturational processes or the effect of familiarization with the test during the second measurements, as has been observed in previous studies (Coledam & De Oliveira, 2020). In contrast, no significant changes were observed in any psychological variables in either active or inactive adolescents, indicating that baseline physical activity level did not moderate the psychological response to the intervention. These findings are consistent with previous studies showing that randomized controlled trials using pedometers had no significant impact on the psychological state of participants (Ferguson et al., 2022). Overall, while baseline physical activity level appeared to play a role in shaping responses in primary outcomes, its influence on secondary outcomes was minimal.

Based on the results obtained, the second research hypothesis (H2) can be partially accepted. The findings indicate that the wearable-based intervention was more effective in adolescent females and in adolescents who were inactive at baseline, particularly with regard to primary outcomes related to body composition. Females showed more consistent and pronounced improvements in fat-related variables, including reductions in fat mass, BMI, and the sum of 3 skinfolds, whereas in males the effects were more limited. Similarly, adolescents with lower baseline physical activity levels exhibited greater improvements in primary outcomes following the intervention, while effects among initially active adolescents were smaller and mainly restricted to fat mass. In contrast, no clear gender- or activity-level–specific effects were observed for secondary outcomes, as changes in physical fitness were modest and psychological variables remained unchanged regardless of subgroup. Taken together, these results suggest that wearable-based interventions promoted through physical education classes may be beneficial for specific subgroups (females and inactives), but their impact on primary and secondary outcomes appears to be context-dependent and moderated by baseline characteristics.

### 4.5. Future lines of research

After the completion of the present study, the following future research lines are proposed: a) future studies should assess whether maturational status influences changes in body composition, physical fitness, and psychological outcomes following wearable-based interventions, given the potential confounding role of growth and pubertal development during adolescence; b) further research is needed to examine whether the specific content and pedagogical approach of physical education classes modulate the effectiveness of wearable-based interventions, particularly in adolescents who are overweight or obese and have low baseline physical activity levels; c) future interventions should explore the use of different wearable devices and platforms to determine whether variations in feedback, usability, and data visualization influence adherence and health-related outcomes; d) in light of the limited effects observed on psychological variables and the low adherence rates, future research should explicitly incorporate gamification strategies (e.g., challenges, rewards, goal-setting, progression systems) and social support mechanisms (e.g., peer interaction, cooperative or competitive group challenges, and teacher or family involvement) to enhance motivation, engagement, and sustained participation; and e) longer follow-up periods are warranted to examine whether wearable-based interventions can produce sustained changes in physical activity behavior, body composition, physical fitness, and psychological well-being over time and to determine whether short-term improvements translate into meaningful long-term health benefits.

### 4.6. Limitations

This research is not without limitations. First, a non-probability convenience sample was used, which, although necessary to recruit overweight and obese adolescents within the school setting, limits the generalizability of the findings to other populations. Second, randomization by class clusters represents a methodological limitation, as cluster randomization can theoretically introduce baseline imbalances between groups and a risk of contamination. However, in this study, cluster randomization was necessary to control for differences in physical activity practices during physical education classes. Moreover, baseline analyses showed no significant differences between the EG and CG in any variable, suggesting that any potential impact on the outcomes was minimal. Third, participants in the EG who completed the intervention received a bonus in their final physical education grade. Although this incentive was intended to enhance engagement and reduce attrition, it may have influenced participants’ motivation and adherence behavior, potentially introducing a source of motivational bias. Therefore, the observed effects should be interpreted with caution, as they may partly reflect extrinsic motivation rather than solely the impact of the wearable-based intervention. Fourth, although the Bonferroni correction was applied to account for multiple comparisons, the large number of statistical tests conducted in this study may still increase the risk of type I error. Therefore, some of the statistically significant results should be interpreted with caution, as they could reflect chance rather than true effects. Fifth, although the intervention duration was optimal and the most commonly used in previous studies involving technological devices, it may be too short to achieve changes in certain parameters of physical fitness and body composition, so longer term studies should be considered. And sixth, although the influence of certain variables was controlled for, maturational status was not considered, which could be affecting changes in body composition and physical fitness in adolescents.

In addition, the external validity of the present findings should be considered with caution. The intervention was conducted in a single secondary school in Spain, which may limit the generalizability of the results to other educational contexts, regions, or cultural settings. School-level characteristics such as socioeconomic background, institutional support for physical activity, teacher involvement, and access to technological resources may have influenced both adherence and intervention effectiveness. Therefore, the observed effects may not be directly transferable to schools with different structural or cultural characteristics. Future studies should aim to replicate these findings across multiple schools, regions, and countries, using more diverse samples, in order to strengthen the external validity and applicability of wearable-based interventions promoted from school settings.

## 5. Conclusion

A 12-week wearable-based intervention promoted through the physical education curriculum may contribute to improvements in body composition, particularly in fat-related variables, and to modest gains in abdominal muscular endurance among adolescents who are overweight or obese. However, these effects were not uniform across the sample and were strongly influenced by factors such as adherence, duration of exposure to the intervention, and baseline characteristics. In particular, females and adolescents who were inactive at baseline appeared to derive greater benefits, suggesting that wearable-based interventions may be especially relevant for specific subgroups within this population.

The findings also indicate that, while wearable devices can support increases in movement volume, their impact on broader physical fitness and psychological well-being may be limited. From an applied perspective, the integration of wearable technologies within the physical education context represents a promising strategy to engage “hard-to-reach” populations such as adolescents who are overweight or obese, who often face greater barriers to regular physical activity. However, given the inconclusive results observed in some variables, the low full-completion rate, and the single-school design, the present results should be interpreted as preliminary. Further research is needed to replicate these findings in larger and more diverse samples, to improve adherence through enhanced intervention design, and to examine whether longer follow-up periods and higher intensity activity targets lead to more consistent and sustained health benefits.

## Supporting information

10.1017/wtc.2026.10039.sm001 Mateo-Orcajada et al. supplementary material 1 Mateo-Orcajada et al. supplementary material

10.1017/wtc.2026.10039.sm002 Mateo-Orcajada et al. supplementary material 2 Mateo-Orcajada et al. supplementary material

## Data Availability

Data can be made available to interested researchers upon request by email to the corresponding author.

## Supplementary material

The supplementary material for this article can be found at http://doi.org/10.1017/wtc.2026.10039.
