# Supplementary material for: Effects of a wearable-based intervention in overweight and obese adolescents: A randomized controlled trial considering gender, baseline activity, and intervention exposure
Source: Wearable Technol. 2026 Mar 30;7:e3. doi: 10.1017/wtc.2026.10039 (PMC13071848; doi:10.1017/wtc.2026.10039)
Supplement: Mateo-Orcajada et al. supplementary material 1 — Mateo-Orcajada et al. supplementary material [file S2631717626100395sup001.docx]

**2.5.1 Questionnaires**

The “Physical Activity Questionnaire for Adolescents” (PAQ-A) was used to assess perceived physical activity levels. This questionnaire has been previously validated in Spanish and demonstrates an intraclass correlation coefficient of 0.71 for the final score (Martínez-Gómez et al., 2009). It consists of nine items, the first eight of which assess the adolescent’s physical activity over the past seven days, while the ninth item identifies whether any condition prevented normal physical activity during that period. The first eight items are answered using a 5-point Likert scale (1: low physical activity level; 5: high physical activity level), and the ninth item is answered dichotomously (yes or no). The arithmetic mean of the first eight items reflects the overall physical activity level, allowing for the classification of adolescents as active (>2.75) or inactive (≤2.75) (Benítez-Porres, 2016).

Life satisfaction was assessed using the “Satisfaction with Life Scale” (SWLS) (Diener, Emmons, Larsen, & Griffin, 1985), an instrument validated in Spanish that demonstrates a good internal consistency (α=0.84). This scale consists of five items scored on a 5-point Likert scale, with total scores ranging from 5 to 25 points, where higher scores indicate greater life satisfaction (Atienza, Pons, Balaguer, & García-Merita, 2000).

Satisfaction of the basic psychological needs (competence, autonomy, and relatedness) was assessed using the Basic Psychological Needs Satisfaction scale (BPNS scale) (Wilson, Rogers, Rodgers, & Wild, 2006). This scale demonstrates a high external validity and internal consistency (competence: α=0.80; autonomy: α=0.69; relatedness: α=0.73) (Moreno-Murcia, Marzo, Martínez-Galindo, & Conte, 2011). It consists of 18 items, six per dimension, scored on a 6-point Likert scale, with minimum and maximum scores for each dimension ranging from 6 to 36, respectively. Higher scores in each dimension indicate a greater satisfaction of that psychological need (Wilson et al., 2006).

**2.5.2 Kinanthropometric and body composition measurement**

Two measurements of each variable were taken, and a third measurement was performed if the difference between the first two exceeded 1% for basic measurements and girths, or 5% for skinfolds. When two measurements were taken, their mean was used as the final value, while the median was used when three measurements were required (F. Esparza-Ros & Vaquero-Cristóbal, 2023; Francisco Esparza-Ros & Vaquero-Cristóbal, 2025).

Validated and reliable equipment was used for all measurements, calibrated prior to each session. For girths, a non-extensible Lufkin W606 PM tape measure (Lufkin, Missouri) with a precision of 0.1 cm was employed; skinfolds were measured using a Harpenden caliper (Burgess Hill, UK) with a precision of 0.2 mm; body mass was assessed using a TANITA DC 430-SMA scale (TANITA, Tokyo) with a precision of 100 g; and height was measured using a SECA 213 stadiometer (SECA, Hamburg) with a precision of 0.1 cm.

**2.5.3 Physical fitness test**

Cardiorespiratory fitness was assessed using the 20-m shuttle run test (Léger, Mercier, Gadoury, & Lambert, 1988). This test consists of running back and forth over a 20-meter distance as many times as possible, following an audio signal that indicates an incremental pace. The test ends when the participant reaches exhaustion or fails to reach the designated line on two consecutive occasions before the audio signal. This test has been shown to be valid and reliable for measuring cardiorespiratory fitness in adolescent populations (Tomkinson, Lang, Blanchard, Léger, & Tremblay, 2019), and allows estimating maximal oxygen consumption (VO₂ max.) using the last completed stage and Léger’s formula (Léger et al., 1988).

Upper-body strength was assessed using two tests: handgrip strength [41] and push-ups (Castro-Piñero et al., 2010). For the handgrip strength test, the participants were instructed to apply their maximum force on a Takei Tkk5401 hand dynamometer (Takei Scientific Instruments, Tokyo, Japan). According to previous research (España-Romero et al., 2010), the optimal position for exerting maximal force was with the elbow fully extended. In the push-up test, participants began in a prone position, with toes in contact with the ground and hands placed shoulder-width apart. From this position, they performed as many push-ups as possible within one minute, keeping the trunk and legs fully extended, bending the elbows to 90°, and then fully extending them to return to the starting position. The test ended when the one-minute period elapsed or when the participants were no longer able to fully extend their arms during the repetitions (Castro-Piñero et al., 2010).

Lower-body power was assessed using the countermovement jump (CMJ) test. This test involves performing a vertical jump as high as possible. Adolescents began the test by standing on a force platform with a sampling frequency of 200 Hz (MuscleLab, Stathelle, Norway), with feet hip-width apart and hands placed on the hips. From this position, they performed a rapid knee flexion to 90°, immediately followed by a full extension of the knees to execute the vertical jump. During the jump, the participants were required to keep their back straight, knees and ankles fully extended, and hands on the hips throughout the movement (Barker, Harry, & Mercer, 2018).

Abdominal muscle endurance was assessed using the curl-up test. Participants began in a supine position, with knees bent at 90° and feet flat on the floor. Arms were crossed over the chest, and during each repetition, the goal was to touch the knees with the elbows, lifting only the scapular area off the mat. The duration of the test was one minute, during which the participants were required to perform the maximum number of repetitions possible (Garcia-Pastor, Salinero, Sanz-Frias, Pertusa, & Del Coso, 2016).

Maximal sprint speed was assessed using a 20-meter sprint test. For this test, the participants started from a standing position on the starting line, and began the sprint at maximal speed at a self-selected moment, without any prior momentum (Bastida Castillo, Gómez Carmona, Pino Ortega, & de la Cruz Sánchez, 2017). Sprint speed was measured using single-beam photocells (Polifemo Light Microgate, Italy) placed at hip height, as this position results in only a 4% likelihood of the light beam being interrupted by arm movement, as compared to a 60% likelihood when placed at chest height (Altmann et al., 2017; Cronin & Templeton, 2008).

**2.6 Procedure**

The same measurement protocol was performed both prior to (pre-test) and following (post-test) the intervention. First, the students completed the PAQ-A, KIDMED, SWLS, and BPNS questionnaires. Subsequently, anthropometric and body composition measurements were taken. After completing these assessments, the participants performed a five-minute progressive warm-up focusing on the main joints involved in the upcoming physical tests. Following the warm-up, adolescents were instructed on the correct execution of the physical tests, and completed a familiarization session with the tests (handgrip strength, CMJ, 20-m sprint, curl-up, and push-up). After familiarization, the students performed two repetitions of each test, with two minutes of rest between repetitions of the same test and five minutes between different tests. This protocol followed previous research recommendations to ensure full recovery and to minimize the fatigue-induced interference on performance in subsequent tests (Coburn & Malek, 2014). After completing two repetitions of each test, a single repetition of the 20-m shuttle run test was performed, as it is a maximal test that leads to exhaustion. The order of the physical tests followed the guidelines set by the National Strength and Conditioning Association (NSCA) (Coburn & Malek, 2014). For tests with two repetitions, the best value was used for analysis. This protocol has been previously used in adolescent populations (Mateo-Orcajada, Abenza-Cano, & Vaquero-Cristóbal, 2025).
