# Supplementary material for: Effects of a wearable-based intervention in overweight and obese adolescents: A randomized controlled trial considering gender, baseline activity, and intervention exposure
Source: Wearable Technol. 2026 Mar 30;7:e3. doi: 10.1017/wtc.2026.10039 (PMC13071848; doi:10.1017/wtc.2026.10039)
Supplement: Mateo-Orcajada et al. supplementary material 2 — Mateo-Orcajada et al. supplementary material [file S2631717626100395sup002.docx]

Table S1. Baseline differences between the control and experimental groups in the study variables.

| Variable | CG | EG | Mean diff. | t | 95% CI Diff. | p |
| --- | --- | --- | --- | --- | --- | --- |
| Primary outcomes | | | | | | |
| Physical activity | 2.69±0.68 | 2.61±0.70 | 0.08 | 0.39 | -0.33; 0.49 | 0.700 |
| Body Mass (kg) | 71.79±10.36 | 72.51±10.78 | -0.72 | -0.23 | -6.92; 5.49 | 0.817 |
| BMI (kg/m^2^) | 27.94±2.40 | 27.78±2.60 | 0.16 | 0.22 | -1.31; 1.63 | 0.826 |
| Waist girth (cm) | 84.96±7.00 | 82.20±7.67 | 2.76 | 1.28 | -1.60; 7.13 | 0.209 |
| Hip girth (cm) | 101.24±8.70 | 102.70±5.12 | -1.46 | -0.82 | -5.06; 2.14 | 0.419 |
| Corrected arm girth (cm) | 23.66±2.79 | 23.49±2.75 | 0.16 | 0.20 | -1.48; 1.81 | 0.843 |
| Corrected thigh girth (cm) | 42.68±4.91 | 43.17±4.15 | -0.49 | -0.37 | -3.19; 2.21 | 0.716 |
| Corrected calf girth (cm) | 28.74±3.72 | 29.21±2.85 | -0.47 | -0.49 | -2.44; 1.50 | 0.630 |
| Fat mass (%) | 39.59±11.34 | 40.16±9.15 | -0.57 | -0.19 | -6.69; 5.56 | 0.853 |
| Muscle mass (kg) | 20.84±4.98 | 20.36±4.91 | 0.48 | 0.33 | -2.46; 3.42 | 0.744 |
| Sum of 3 skinfolds | 92.35±29.92 | 95.14±22.35 | -2.79 | -0.36 | -18.48; 12.91 | 0.722 |
| Secondary outcomes | | | | | | |
| Life satisfaction | 16.83±4.06 | 17.22±6.53 | -0.39 | -0.24 | -3.62; 2.84 | 0.808 |
| Competence | 23.91±7.18 | 22.83±9.55 | 1.09 | 0.44 | -3.93; 6.11 | 0.665 |
| Autonomy | 23.96±5.59 | 22.13±8.39 | 1.83 | 0.87 | -2.41; 6.06 | 0.390 |
| Relatedness | 21.39±5.94 | 25.20±9.33 | -3.80 | -1.65 | -8.45; 0.84 | 0.106 |
| VO_2_ max. (ml/kg/min) | 35.12±4.21 | 35.23±2.61 | -0.11 | -0.11 | -2.19; 1.97 | 0.914 |
| Handgrip right hand (kg) | 26.85±8.85 | 26.12±7.80 | 0.72 | 0.30 | -4.19; 5.64 | 0.768 |
| Handgrip left hand (kg) | 24.92±6.64 | 24.50±7.35 | 0.42 | 0.20 | -3.69; 4.53 | 0.839 |
| CMJ (cm) | 18.02±6.55 | 17.63±5.35 | 0.39 | 0.22 | -3.14; 3.91 | 0.826 |
| 20 m sprint (s) | 4.37±0.49 | 4.14±0.97 | 0.22 | 1.00 | -0.23; 0.67 | 0.322 |
| Curl-up (reps) | 16.92±11.69 | 14.13±8.93 | 2.79 | 0.92 | -3.34; 8.91 | 0.365 |
| Push-up (reps) | 2.61±5.25 | 1.26±4.28 | 1.35 | 0.96 | -1.50; 4.19 | 0.345 |

BMI: body mass index; VO2 max: maximal oxygen consumption; CMJ: countermovement jump.

Table S2. Differences in the study variable between the pre and posttest in the experimental and control groups.

| Variable | Group | Timepoint | | | | | | | Wearable use * gender | | | Wearable use * PA level | | |
| --- | --- | --- | --- | --- | --- | --- | --- | --- | --- | --- | --- | --- | --- | --- |
|  |  | Pre | Post | Pre - post diff. | F | p | 95% CI diff. | η2 | F | p | 95% CI diff. | F | p | 95% CI diff. |
| Primary outcomes | | | | | | | | | | | | | | |
| Physical activity | CG | 2.69±0.68 | 2.70±0.61 | -0.01±0.09 | 0.01 | 0.920 | -0.20; 0.18 | 0.00 | 0.39 | 0.538 | -0.24; 0.13 | 0.04 | 0.846 | -0.19;0.16 |
|  | EG | 2.61±0.70 | 2.80±0.62 | -0.19±0.09 | 4.16 | 0.048 | -0.38; -0.00 | 0.09 | 2.66 | 0.110 | -0.33; 0.04 | 4.42 | 0.041 | -0.36; -0.01 |
| Body  Mass (kg) | CG | 71.93±10.50 | 73.07±11.70 | -1.15±0.55 | 4.27 | 0.045 | -2.26; -0.03 | 0.10 | 2.57 | 0.117 | -2.02;0.23 | 4.17 | 0.048 | -2.28; -0.01 |
|  | EG | 72.51±10.78 | 72.47±11.28 | 0.05±0.50 | 0.01 | 0.928 | -0.97;1.06 | 0.00 | 0.10 | 0.752 | -1.18;0.86 | 0.01 | 0.928 | -0.99;1.08 |
| BMI (kg/m^2^) | CG | 27.99±2.44 | 28.07±2.80 | -0.08±0.21 | 0.13 | 0.720 | -0.50;0.35 | 0.00 | 0.04 | 0.844 | -0.49;0.40 | 0.14 | 0.714 | -0.51;0.35 |
|  | EG | 27.78±2.60 | 27.23±2.56 | 0.55±0.19 | 8.14 | 0.007 | 0.16;0.94 | 0.17 | 6.85 | 0.013 | 0.12;0.93 | 8.03 | 0.007 | 0.16;0.94 |
| Waist girth (cm) | CG | 85.28±7.61 | 85.22±7.99 | 0.06±0.55 | 0.01 | 0.913 | -1.05;1.17 | 0.00 | 0.03 | 0.874 | -1.07;1.25 | 0.03 | 0.862 | -1.01;1.20 |
|  | EG | 82.20±7.67 | 81.84±8.33 | 0.36±0.49 | 0.55 | 0.461 | -0.62;1.34 | 0.01 | 0.45 | 0.507 | -0.68;1.36 | 0.48 | 0.492 | -0.64;1.31 |
| Hip girth (cm) | CG | 101.46±6.00 | 102.75±6.39 | -1.30±0.51 | 6.40 | 0.016 | -2.33; -0.26 | 0.14 | 4.94 | 0.072 | -2.24; -0.11 | 6.18 | 0.017 | -2.34; -0.24 |
|  | EG | 102.70±5.12 | 102.19±5.16 | 0.50±0.45 | 1.23 | 0.274 | -0.41; 1.42 | 0.03 | 0.77 | 0.385 | -0.53;1.35 | 1.18 | 0.284 | -0.43;1.43 |
| Corrected arm girth (cm) | CG | 24.13±2.96 | 24.19±2.82 | -0.06±0.29 | 0.04 | 0.839 | -0.64;0.52 | 0.00 | 0.35 | 0.558 | -0.76;0.41 | 0.05 | 0.827 | -0.65;0.52 |
|  | EG | 23.49±2.75 | 23.59±2.50 | -0.91±0.25 | 0.13 | 0.720 | -0.60;0.42 | 0.00 | 0.00 | 0.990 | -0.52;0.52 | 0.12 | 0.735 | -0.61;0.43 |
| Corrected thigh girth (cm) | CG | 43.67±4.74 | 44.38±4.13 | -0.72±0.70 | 1.06 | 0.309 | -2.13;0.69 | 0.03 | 1.02 | 0.320 | -2.21;0.74 | 0.96 | 0.334 | -2.09;0.73 |
|  | EG | 43.17±4.15 | 43.15±4.45 | 0.20±0.62 | 0.00 | 0.975 | -1.23;1.27 | 0.00 | 0.00 | 0.960 | -1.27;1.33 | 0.00 | 0.988 | -1.26;1.24 |
| Corrected calf girth (cm) | CG | 29.67±3.43 | 30.19±2.95 | -0.52±0.32 | 2.64 | 0.112 | -1.17;0.13 | 0.06 | 2.68 | 0.110 | -1.23;0.13 | 2.97 | 0.093 | -1.19;0.10 |
|  | EG | 29.21±2.85 | 29.68±2.86 | -0.47±0.28 | 2.70 | 0.109 | -1.04;0.11 | 0.07 | 2.29 | 0.139 | -1.04;0.15 | 2.56 | 0.118 | -1.01;0.12 |
| Fat mass (%) | CG | 35.11±5.70 | 34.62±6.13 | 0.49±0.32 | 2.36 | 0.131 | -0.15;1.14 | 0.05 | 1.73 | 0.195 | -0.23;1.09 | 2.30 | 0.136 | -0.16;1.14 |
|  | EG | 34.90±5.08 | 32.95±4.57 | 1.96±0.33 | 35.86 | <0.001 | 1.30;2.62 | 0.44 | 36.65 | <0.001 | 1,35;2.69 | 35.62 | <0.001 | 1.30;2.62 |
| Muscle mass (kg) | CG | 20.84±4.98 | 23.24±4.24 | 0.61±0.32 | 3.58 | 0.065 | -0.04; 1.25 | 0.08 | 2.59 | 0.115 | -0.13;1.19 | 3.58 | 0.065 | -0.04;1.25 |
|  | EG | 20.36±4.91 | 20.51±4.77 | -0.15±0.32 | 0.21 | 0.647 | -0.79;0.50 | 0.01 | 0.04 | 0.836 | -0.73;0.59 | 0.21 | 0.647 | -0.79;0.50 |
| Sum of 3 skinfolds | CG | 89.83±28.00 | 91.53±27.48 | -1.70±1.99 | 0.73 | 0.398 | -5.71;2.31 | 0.02 | 0.25 | 0.620 | -5.02;3.02 | 0.72 | 0.402 | -5.76;2.35 |
|  | EG | 95.14±22.35 | 88.76±20.94 | 6,38±1.94 | 10.78 | 0.002 | 2.46;10.30 | 0.20 | 8.62 | 0.005 | 1.79;9.64 | 10.55 | 0.002 | 2.42;10.35 |
| Secondary outcomes | | | | | | | | | | | | | | |
| Life satisfaction | CG | 16.83±4.06 | 16.35±5.47 | 0.48±1.47 | 0.11 | 0.747 | -2.49;3.44 | 0.00 | 0.07 | 0.791 | -2.65;3.46 | 0.09 | 0.761 | -2.54;3.45 |
|  | EG | 17.22±6.53 | 17.70±5.71 | -0.48±1.47 | 0.11 | 0.747 | -3.44;2.49 | 0.00 | 0.07 | 0.791 | -3.46;2.65 | 0.09 | 0.761 | -3.45;2.54 |
| Competence | CG | 23.91±7.18 | 22.78±8.01 | 1.13±1.87 | 0.37 | 0.548 | -2.63;4.89 | 0.01 | 0.28 | 0.597 | -2.85;4.90 | 0.34 | 0.566 | -2.70;4.87 |
|  | EG | 22.83±9.55 | 25.78±8.43 | -2.96±1.87 | 2.51 | 0.120 | -6.72;0.81 | 0.05 | 2.20 | 0.145 | -6.72;1.02 | 2.41 | 0.128 | -6.70;0.87 |
| Autonomy | CG | 23.96±5.59 | 23.65±7.48 | 0.30±1.96 | 0.24 | 0.877 | -3.64;4.24 | 0.00 | 0.01 | 0.911 | -3.83;4.29 | 0.02 | 0.896 | -3.71;4.22 |
|  | EG | 22.13±8.39 | 24.83±8.30 | -2.70±1.96 | 1.90 | 0.175 | -6.64;1.24 | 0.04 | 1.69 | 0.200 | -6.68;1.44 | 1.82 | 0.185 | -6.62;1.31 |
| Relatedness | CG | 21.39±5.94 | 20.52±8.37 | 0.87±1.85 | 0.22 | 0.640 | -2.85;4.59 | 0.01 | 0.23 | 0.637 | -2.93;4.74 | 0.20 | 0.659 | -2.92;4.56 |
|  | EG | 25.20±9.33 | 26.57±7.24 | -1.37±1.85 | 0.55 | 0.462 | -5.09;2,35 | 0.01 | 0.54 | 0.465 | -5.24;2.43 | 0.51 | 0.479 | -5.06;2.42 |
| VO_2_ max.  (ml/kg/min) | CG | 35.12±3.87 | 34.87±4.25 | 0.24±0.55 | 0.20 | 0.659 | -0.86;1.35 | 0.01 | 0.18 | 0.676 | -0.90;1.38 | 0.19 | 0.668 | -0.88;1.36 |
|  | EG | 35.23±2.61 | 34.77±2.82 | 0.46±0.52 | 0.78 | 0.385 | -0.60;1.51 | 0.02 | 0.74 | 0.394 | -0.62;1.55 | 0.77 | 0.387 | -0.61;1.53 |
| Handgrip right hand (kg) | CG | 26.85±8.85 | 27.58±9.10 | -0.73±1.43 | 0.26 | 0.614 | -3.62;2.16 | 0.01 | 0.53 | 0.469 | -4.00;1.87 | 0.27 | 0.603 | -3.65;2.14 |
|  | EG | 26.12±7.80 | 24.46±7.92 | 1.66±1.47 | 1.29 | 0.263 | -1.29;4.61 | 0.03 | 1.82 | 0.184 | -0.99;5.01 | 1.32 | 0.257 | -1.27;4.64 |
| Handgrip left hand (kg) | CG | 24.92±6.64 | 25.55±7.78 | -0.63±0.48 | 1.75 | 0.192 | -1.59;0.33 | 0.04 | 2.25 | 0.140 | -1.70;0.25 | 1.80 | 0.187 | -1.59;0.32 |
|  | EG | 24.50±7.35 | 24.39±6.38 | 0.11±0.49 | 0.05 | 0.818 | -0.87;1.09 | 0.00 | 0.19 | 0.666 | -0.78;1.21 | 0.06 | 0.804 | -0.86;1.10 |
| CMJ (cm) | CG | 18.66±5.51 | 19.83±5.39 | -0.18±1.20 | 0.96 | 0.332 | -3.60;1.24 | 0.02 | 1.17 | 0.286 | -3.80;1.15 | 0.95 | 0.336 | -3.63;1.27 |
|  | EG | 17.63±5.35 | 17.57±7.10 | 0.06±1.17 | 0.00 | 0.959 | -2.31;2.43 | 0.00 | 0.03 | 0.866 | -2.22;2.62 | 0.00 | 0.957 | -2.33;2.46 |
| 20 m sprint (s) | CG | 4.37±0.49 | 4.29±0.47 | 0.07±0.18 | 0.16 | 0.691 | -0.29;0.44 | 0.00 | 0.01 | 0.930 | -0.35;0.39 | 0.15 | 0.703 | -0.30;0.44 |
|  | EG | 4.14±0.97 | 4.08±0.94 | 0.06±0.19 | 0.11 | 0.742 | -0.31;0.44 | 0.00 | 0.42 | 0.521 | -0.26;0.50 | 0.12 | 0.703 | -0.31;0.44 |
| Curl-up (reps) | CG | 16.92±11.68 | 20.79±13.09 | -3.88±1.67 | 5.48 | 0.024 | -7.21; -0.54 | 0.11 | 5.36 | 0.025 | -7.39; -0.51 | 5.36 | 0.025 | -7.25; -0.50 |
|  | EG | 14.13±8.93 | 19.74±7.57 | -5.61±1.69 | 11.01 | 0.002 | -9.01; -2.20 | 0.20 | 10.05 | 0.003 | -9.05; -2.01 | 10.76 | 0.002 | -9.06; -2.16 |
| Push-up (reps) | CG | 2.61±5.25 | 2.61±4.31 | 0.00±0.57 | 0.00 | 1.000 | -1.14;1.14 | 0.00 | 0.05 | 0.822 | -1.29;1.03 | 0.00 | 1.000 | -1.15;1.15 |
|  | EG | 1.26±4.28 | 1.09±4.80 | 0.17±0.57 | 0.10 | 0.760 | -0.97;1.31 | 0.00 | 0.28 | 0.599 | -0.85;1.46 | 0.09 | 0.761 | -0.97;1.32 |

BMI: body mass index; VO2 max: maximal oxygen consumption; CMJ: countermovement jump.

Table S3. Changes between the experimental and control groups after the intervention in the study variables.

| Variable | Wearable use | | | | |
| --- | --- | --- | --- | --- | --- |
|  | Pre-Post CG – Pre-post EG | F | p | 95%CI diff. | η2 |
| Primary outcomes | | | | | |
| Physical activity | 0.24±0.16 | 2.27 | 0.141 | -0.08; 0.56 | 0.06 |
| Body mass (Kg) | -1.94±0.72 | 7.21 | 0.011 | -3.40; -0.47 | 0.17 |
| BMI (kg/m^2^) | -0.65±0.31 | 4.44 | 0.042 | -1.27; -0.30 | 0.11 |
| Waist girth (cm) | -0.54±0.79 | 0.48 | 0.495 | -2.14;1.05 | 0.01 |
| Hips girth (cm) | -2.01±0.73 | 7.49 | 0.010 | -3.49; -0.52 | 0.17 |
| Corrected arm girth (cm) | 0.01±0.42 | 0.00 | 0.974 | -0.83;0.86 | 0.00 |
| Corrected thigh girth (cm) | -0.88±1.02 | 0.75 | 0.392 | -2.94;1.18 | 0.02 |
| Corrected calf girth (cm) | -0.13±0.47 | 0.07 | 0.790 | -1.07;0.82 | 0.00 |
| Fat mass (%) | -1.55±0.56 | 7.58 | 0.009 | -2.70; -0.41 | 0.14 |
| Muscle mass (kg) | 0.46±0.31 | 2.14 | 0.152 | -0.18;1.09 | 0.06 |
| Sum of 3 skinfolds | -8.58±3.36 | 6.54 | 0.015 | -15.39; -1.77 | 0.15 |
| Secondary outcomes | | | | | |
| Life satisfaction | 0.41±2.43 | 0.03 | 0.866 | -4.52;5.34 | 0.00 |
| Competence | 2.82±3.02 | 0.88 | 0.355 | -3.29;8.94 | 0.02 |
| Autonomy | 1.70±3.14 | 0.29 | 0.592 | -4.67;8.06 | 0.01 |
| Relatedness | 2.30±2.97 | 0.60 | 0.443 | -3.72;8.33 | 0.02 |
| VO_2_ max. (ml/kg/min) | 0.01±0.89 | 0.00 | 0.988 | -1.78;1.81 | 0.00 |
| Handgrip right hand (kg) | -1.46±2.55 | 0.33 | 0.570 | -6.63;3.71 | 0.01 |
| Handgrip left hand (kg) | -0.09±0.75 | 0.01 | 0.910 | -1.61;1.44 | 0.00 |
| CMJ (cm) | -1.44±2.03 | 0.50 | 0.483 | -5.56;2.68 | 0.01 |
| 20-m sprint | 0.03±0.33 | 0.01 | 0.940 | -0.65;0.70 | 0.00 |
| Curl-up (reps) | 1.41±2.21 | 0.41 | 0.528 | -3.07;5.89 | 0.01 |
| Push-up (reps) | -0.84±0.61 | 1.91 | 0.175 | -2.07;0.39 | 0.05 |

BMI: body mass index; VO2 max: maximal oxygen consumption; CMJ: countermovement jump.

Table S4. Pre–post changes in physical activity, body composition, psychological and physical fitness variables according to intervention exposure length.

| Variable | Exposure | Pre | Post | Pre - post diff. | p | 95% CI diff. | η2 |
| --- | --- | --- | --- | --- | --- | --- | --- |
| Primary outcomes | | | | | | | |
| Physical activity | Long | 2.52±0.59 | 2.86±0.59 | -0.33 | 0.032 | -0.69; -0.03 | 0.25 |
|  | Short | 2.65±0.78 | 2.76±0.67 | -0.10 | 0.468 | -0.39; 0.19 | 0.03 |
| Body  Mass (kg) | Long | 74.12±13.11 | 73.74±13.26 | 0.37 | 0.606 | -1.10; 1.85 | 0.01 |
|  | Short | 71.48±9.38 | 71.64±10.27 | -0.16 | 0.776 | -1.35; 1.02 | 0.00 |
| BMI (kg/m^2^) | Long | 27.70±2.92 | 27.28±2.76 | 0.42 | 1.000 | -0.92; 1.05 | 0.00 |
|  | Short | 27.83±2.48 | 27.23±2.56 | 0.60 | 0.961 | -1.18; 1.27 | 0.01 |
| Waist girth (cm) | Long | 81.12±7.63 | 80.47±9.61 | 0.65 | 0.464 | -1.15; 2.44 | 0.03 |
|  | Short | 82.89±7.90 | 82.71±7.65 | 0.18 | 0.798 | -1.26; 1.62 | 0.00 |
| Hip girth (cm) | Long | 103.59±6.08 | 102.78±6.03 | 0.82 | 0.335 | -0.90; 2.54 | 0.04 |
|  | Short | 102.12±4.54 | 101.82±4.72 | 0.30 | 0.656 | -1.08; 1.68 | 0.01 |
| Corrected arm girth (cm) | Long | 23.27±3.05 | 24.08±2.54 | -0.82 | 0.051 | -1.64; 0.00 | 0.17 |
|  | Short | 23.64±2.64 | 23.27±2.52 | 0.38 | 0.249 | -0.28; 1.03 | 0.06 |
| Corrected thigh girth (cm) | Long | 43.15±5.28 | 45.02±4.46 | -1.87 | 0.081 | -3.99; 0.25 | 0.14 |
|  | Short | 43.18±3.45 | 41.95±4.17 | 1.23 | 0.147 | -0.47; 2.94 | 0.10 |
| Corrected calf girth (cm) | Long | 29.90±2.32 | 31.11±1.97 | -1.21 | 0.058 | -2.28; 0.14 | 0.17 |
|  | Short | 28.77±3.15 | 28.75±3.02 | 0.02 | 0.972 | -0.84; 0.87 | 0.00 |
| Fat mass (%) | Long | 38.33±6.26 | 34.20±6.52 | 4.13 | 0.015 | 0.88; 7.38 | 0.25 |
|  | Short | 41.33±10.67 | 40.67±8.91 | 0.67 | 0.600 | -1.94; 3.27 | 0.01 |
| Muscle mass (kg) | Long | 20.00±6.12 | 20.54±5.83 | -0.53 | 0.144 | -1.27; 0.20 | 0.10 |
|  | Short | 20.59±4.19 | 20.49±4.19 | 0.10 | 0.724 | -0.49; 0.69 | 0.01 |
| Sum of 3 skinfolds | Long | 93.67±18.58 | 81.78±17.00 | 11.89 | <0.001 | 5.71; 18.07 | 0.43 |
|  | Short | 96.08±25.11 | 93.24±22.56 | 2.84 | 0.247 | -2.12; 7.80 | 0.06 |
| Secondary outcomes | | | | | | | |
| Life satisfaction | Long | 18.00±7.79 | 18.89±4.78 | -0.89 | 0.773 | -7.22; 5.44 | 0.00 |
|  | Short | 16.71±5.85 | 16.93±6.28 | -0.21 | 0.931 | -5.29; 4.86 | 0.00 |
| Competence | Long | 22.67±10.11 | 25.56±7.57 | -2.89 | 0.463 | -10.93; 5.15 | 0.03 |
|  | Short | 22.93±9.56 | 25.93±9.21 | -3.00 | 0.344 | -9.45; 3.45 | 0.04 |
| Autonomy | Long | 21.67±9.53 | 26.89±6.41 | -5.22 | 0.201 | -13.45; 3.00 | 0.08 |
|  | Short | 22.43±7.94 | 23.50±9.30 | -1.07 | 0.739 | -7.66; 5.52 | 0.01 |
| Relatedness | Long | 26.33±10.82 | 28.11±5.78 | -1.79 | 0.620 | -9.11; 5.56 | 0.01 |
|  | Short | 24.46±8.59 | 25.57±8.09 | -1.11 | 0.699 | -6.99; 4.77 | 0.01 |
| VO_2_ max.  (ml/kg/min) | Long | 35.71±2.14 | 35.29±3.14 | 0.43 | 0.664 | -1.59; 2.45 | 0.01 |
|  | Short | 34.92±2.91 | 34.44±2.66 | 0.48 | 0.547 | -1.15; 2.10 | 0.02 |
| Handgrip right hand (kg) | Long | 27.62±6.73 | 27.09±8.69 | 0.53 | 0.882 | -0.78; 11.84 | 0.04 |
|  | Short | 25.16±8.52 | 25.99±7.29 | -0.83 | 0.737 | -5.89; 4.23 | 0.01 |
| Handgrip left hand (kg) | Long | 25.87±6.13 | 25.19±5.59 | 0.68 | 0.380 | -0.89; 2.25 | 0.04 |
|  | Short | 23.63±8.13 | 23.88±6.99 | -0.25 | 0.683 | -1.51; 1.01 | 0.01 |
| CMJ (cm) | Long | 20.02±2.39 | 18.64±7.76 | 1.38 | 0.580 | -3.72; 6.47 | 0.02 |
|  | Short | 16.10±6.20 | 16.89±6.85 | -0.79 | 0.693 | -4.87; 3.30 | 0.01 |
| 20 m sprint (s) | Long | 4.22±0.28 | 3.69±1.41 | 0.52 | 0.214 | -0.33; 1.37 | 0.07 |
|  | Short | 4.09±1.25 | 4.33±0.34 | -0.24 | 0.481 | -0.92; 0.45 | 0.02 |
| Curl-up (reps) | Long | 11.33±7.45 | 19.33±7.45 | -8.00 | 0.002 | -12.57; -3.43 | 0.39 |
|  | Short | 15.93±9.58 | 20.00±7.91 | -4.07 | 0.031 | -7.73; -0.41 | 0.20 |
| Push-up (reps) | Long | 1.22±0.44 | 1.00±0.57 | 0.22 | 0.674 | -0.86; 1.31 | 0.01 |
|  | Short | 1.93±5.44 | 1.79±6.13 | 0.14 | 0.736 | -0.73; 1.01 | 0.01 |

BMI: body mass index; VO2 max: maximal oxygen consumption; CMJ: countermovement jump.
